# Using Electronic Data Collection Platforms to Assess Complementary and Integrative Health Patient-Reported Outcomes: Feasibility Project

**DOI:** 10.2196/15609

**Published:** 2020-06-26

**Authors:** Jolie N Haun, Amy C Alman, Christine Melillo, Maisha Standifer, Julie McMahon-Grenz, Marlena Shin, W A Lapcevic, Nitin Patel, A Rani Elwy

**Affiliations:** 1 Research Service James A. Haley VA Medical Center Tampa, FL United States; 2 Department of Community & Family Health College of Public Health University of South Florida Tampa, FL United States; 3 Department of Public Health University of South Florida Tampa, FL United States; 4 Department of Pharmacy Practice College of Pharmacy University of South Florida Tampa, FL United States; 5 Center for Healthcare Organization and Implementation Research Veterans Affairs Boston Healthcare System Boston, MA United States; 6 Performance Improvement and Reporting VHA Office of Community Care Department of Veteran Affairs Washington, DC United States; 7 Center for Healthcare Organization and Implementation Research Bedford Veterans Affairs Medical Center Bedford, MA United States; 8 Brown University Providence, RI United States

**Keywords:** integrative medicine, health information technology, health services research, mobile phone, patient-reported outcomes, veteran

## Abstract

**Background:**

The Veteran Administration (VA) Office of Patient-Centered Care and Cultural Transformation is invested in improving veteran health through a whole-person approach while taking advantage of the electronic resources suite available through the VA. Currently, there is no standardized process to collect and integrate electronic patient-reported outcomes (ePROs) of complementary and integrative health (CIH) into clinical care using a web-based survey platform. This quality improvement project enrolled veterans attending CIH appointments within a VA facility and used web-based technologies to collect ePROs.

**Objective:**

This study aimed to (1) determine a practical process for collecting ePROs using patient email services and a web-based survey platform and (2) conduct analyses of survey data using repeated measures to estimate the effects of CIH on patient outcomes.

**Methods:**

In total, 100 veterans from one VA facility, comprising 11 cohorts, agreed to participate. The VA patient email services (Secure Messaging) were used to manually send links to a 16-item web-based survey stored on a secure web-based survey storage platform (Qualtrics). Each survey included questions about patient outcomes from CIH programs. Each cohort was sent survey links via Secure Messaging (SM) at 6 time points: weeks 1 through 4, week 8, and week 12. Process evaluation interviews were conducted with five primary care providers to assess barriers and facilitators to using the patient-reported outcome survey in usual care.

**Results:**

This quality improvement project demonstrated the usability of SM and Qualtrics for ePRO collection. However, SM for ePROs was labor intensive for providers. Descriptive statistics on health competence (2-item Perceived Health Competence Scale), physical and mental health (Patient-Reported Outcomes Measurement Information System Global-10), and stress (4-item Perceived Stress Scale) indicated that scores did not significantly change over time. Survey response rates varied (18/100, 18.0%-42/100, 42.0%) across each of the 12 weekly survey periods. In total, 74 of 100 participants provided ≥1 survey, and 90% (66/74) were female. The majority, 62% (33/53) of participants, who reported the use of any CIH modality, reported the use of two or more unique modalities. Primary care providers highlighted specific challenges with SM and offered solutions regarding staff involvement in survey implementation.

**Conclusions:**

This quality improvement project informs our understanding of the processes currently available for using SM and web-based data platforms to collect ePROs. The study results indicate that although it is possible to use SM and web-based survey platforms for ePROs, automating scheduled administration will be necessary to reduce provider burden. The lack of significant change in ePROs may be due to standard measures taking a biomedical approach to wellness. Future work should focus on identifying ideal ePRO processes that would include standardized, whole-person measures of wellness.

## Introduction

### Background

The Veterans Health Administration (VHA) is committed to increasing the use of health information technology (HIT) to promote personalized and patient-driven health services, including complementary and integrative health (CIH) [1]. The integration of HIT has the potential to increase quality and access to CIH, enhance patient outcomes, increase efficiency, and decrease costs [2,3]. Effective implementation of integrated HIT, such as telehealth and electronic health records, is a priority for the VHA [4].

The VHA prioritizes access to CIH [5,6]. Complementary treatment is based on Eastern medicine philosophies and includes a variety of modalities, including but not limited to yoga, mindfulness, and acupuncture [7]. Integrative health is the use of both Western or *traditional* treatments in combination with *complementary* treatments [8]. Integrative health tends to lower any power differential between patient and provider as well as focus on contextual health [8]. Recent VHA programs focus on improving access to CIH modalities for all veterans, making integration of CIH into health plans a priority within the VHA [1,6].

Within the VHA, HIT for health care delivery is not only beneficial for providers and their delivery of health care services but is also advantageous for meeting patient-specific needs. Using HIT for health care delivery facilitates timely reporting of outcomes (ie, improves recall accuracy) and eliminates the potential for misplaced documentation. The process empowers patients to make informed health care decisions, improves patient satisfaction, and streamlines organizational processes that, historically, were barriers to the delivery of health care services [9]. Electronic health communication systems have improved clinical effectiveness and enhanced communication between patients and providers [10]. Patients managing chronic conditions have reported satisfaction in web-based reporting systems that facilitate effective communication of biomedical metrics (eg, blood pressure and weight) [11]. Recent studies have demonstrated the usability and implementation of HIT platforms to collect electronic patient-reported outcomes (ePROs), such as biomedical metrics for disease management [12-14].

Patient-centered outcome measures for CIH are increasingly important as the use of nontraditional therapies and treatments increases in clinical settings [15]. Several research studies support the advantages of collecting ePROs through mobile technology application, secure messaging (SM), or text messaging [16-18], but there is little published research on the use of HIT to collect CIH-related ePROs within the unique Veteran Administration (VA) system [19]. There is a need to develop a practical process for collecting and integrating ePROs to improve patient care within the VHA [20-22].

### Objectives

This study aimed to (1) determine a practical process for collecting and integrating ePROs using SM and a web-based survey platform and (2) conduct analyses of pilot survey data using repeated measures to estimate the effects of CIH on patient outcomes. This paper provides lessons learned from the implementation of ePRO survey methods within the VA.

## Methods

### Design

This 1-year pilot project engaged veterans attending CIH appointments within one VA facility. We used process documentation, quantitative repeated measures surveys, and qualitative interviews to meet project objectives.

### Veterans Sample

This project used a convenience sample. Project team members reached out to local primary care providers (PCPs; N=21) who were known to make referrals to CIH program services at the project site. These PCPs were identified as early adopters in the CIH program. Early adopter PCPs were asked to provide a list of potential participants who participated in at least one CIH program or activity at the project site based on their personal knowledge of veteran wellness activities.

Of the responsive providers (n=5), a participant pool of 227 veterans was identified. The project site had a robust integrative health program at the time of participant pool identification. Providers often used referral to the integrative health program as a proxy for the inclusion criteria of the CIH program or activity participation. Additionally, at the time of participant pool identification, providers based their list of potential participants on general knowledge of veterans having used CIH program services in the recent past. Between participant pool identification and completion of recruitment, up to four months had passed, generating a subgroup of participants that were no longer participating in CIH programs during the project. Our final convenience sample of 100 veterans was recruited via telephone to participate in the project based on the following inclusion criteria: (1) participated in at least one CIH program or activity at the project site and (2) had access to SM to complete the web-based surveys.

Two electronic messaging platforms for data collection were identified by operational partners and clinical providers: SM, a messaging platform provided to veterans through MyHealtheVet (a veteran-facing health care portal), and ANNIE, a mobile app that allows messaging, alerts, and push notifications, and can send messages to mobile phones that are not smartphones. ANNIE was not accessible for utilization at the time of project implementation. Owing to the unavailability of ANNIE, only the SM web-based platform was used. SM, as a messaging service, requires its own unique set of processes to send individual messages to each veteran in the project. We developed a customized protocol for using SM to collect and integrate ePROs, which was tested in this project.

Participants were grouped into cohorts to facilitate data collection. Each cohort was the result of the project team making telephone calls once per week (typically Friday). Participants who agreed to participate were included in that week’s cohort and notified of the intent of the study, number and frequency of assessments, time burden for each assessment, and the need to access their SM account routinely (typically Monday). Thus, a cohort consisted of veterans who received electronic requests for survey completion at the same time points. The 11 cohorts ranged in size from 2 to 21 participants, with more participants in the initial cohorts and fewer in the latter cohorts, with an average cohort size of 9. Process evaluation interviews were conducted with 5 PCPs involved in the SM implementation of the ePRO survey to assess the acceptability, appropriateness, and feasibility of using SM to direct veterans to the web-based ePRO survey in future VA sites.

### Data Collection Procedure

The effects of CIH activities may change over time and are often measured at variable time points [23]. As such, all participants within a cohort were sent the survey link via SM at 6 time points: weeks 1 through 4, week 8, and week 12. The time points were selected to evaluate both short- (1-4 weeks) and long-term (week 8 and week 12) assessment of ePRO responses. The initial weekly assessments were used not only to capture an initial change in ePROs (based on new participation in CIH activities) but also to test the use of weekly survey links (eg, how to distinguish survey links and reminders, and how to manage data). More than six time points were considered potentially burdensome for the participants. Rolling enrollment and sending of SM links occurred over a 25-week period. Each participant was sent an SM message with a link and a unique personal identification number at the beginning of each week and again 3 days later to nonrespondents. Each link contained a web-based survey consisting 16 items from the scales described in the *Scales* section below as well as items to indicate the types of CIH programs in which the participant was currently participating. Survey data were collected and managed via Qualtrics), a web-based survey platform.

Process evaluation interviews were conducted via telephone. One researcher conducted the interview, and another researcher took extensive notes on this interview, capturing as much verbatim information as possible. Interviews lasted approximately 30 min and focused on capturing stakeholders’ perspectives on the acceptability, appropriateness, and feasibility of using the ePRO survey with veterans receiving CIH services through the SM platform. Our conceptual framework is derived from the categorization of implementation outcomes by Proctor et al [24], the implementation outcomes framework. We specifically assessed (1) acceptability or the extent to which adopting the ePRO survey is agreeable, palatable, or satisfactory among key stakeholders; (2) appropriateness, the perceived fit, relevance, or compatibility of the innovation or evidence-based practice for VA primary care providers and staff; and (3) feasibility or the extent to which the ePRO survey can be successfully used or carried out within VA primary care.

### Scales

Participants completed a 16-item web-based survey consisting of items that measured health competence on the 2-item Perceived Health Competence Scale (PHCS-2), physical and mental health on the 10-item Patient Reported Outcome Measurement Information System (PROMIS-10), and perceived stress on the 4-item Perceived Stress Scale 4 (PSS-4). These scales were chosen to obtain data across a broad range of patient-reported health outcomes, in which CIH modalities may have an impact. Survey links did not have an expiration time designated so they could be completed as assigned or at any point during the project. Demographic data were extracted from the electronic medical records of all participants.

The PHCS-2 is a balanced subscale consisting of 2 questions (1 positively worded and 1 negatively worded) chosen from the larger 8-item PHCS and measures the degree to which an individual feels capable of reasonably managing his or her own health outcomes. The PHCS has previously shown to be a valid and reliable measure of health competence [25]. Total scores range from 2 to 10, with higher scores reflecting increased perceived health competence.

The PROMIS-10 short form consists of 10 items that assess the general domains of physical and mental health and functioning. PROMIS was developed and validated by investigators at the National Institutes of Health to provide clinicians and researchers with accessible item banks to measure patient-reported health status [26,27]. Raw scores for the physical and mental domains ranged between 4 and 20. Higher scores reflect better physical and mental functioning.

The PSS-4 measures the degree to which situations in one’s life over the past month are appraised as stressful. Items were designed to detect how unpredictable, uncontrollable, and overloaded respondents find their lives. PSS-4 was derived from the longer PSS-14 [28]. As the questions are of a general nature and are not directed at any particular subpopulation, using the abbreviated version (or any version) with a diverse population is predicted to yield equally reliable results. The score ranges from 0 to 16, with higher scores representing more stress.

### Data Analysis

Data are presented as mean (SD) for continuous data and frequencies and percentages for categorical data. Chi-square tests and two-tailed *t* tests were used to compare demographics between survey responders and nonresponders. Survey response rates were considered a measure of ePRO collection process feasibility, with a focus on practicality [29]. Survey response rates were defined as the percentage of participants who responded to at least one survey question over the examination period. We also examined the length of time to completion and completeness of responses to the scale items (PHCS-2, PROMIS-10, and PSS-4). Final scores for the scales were calculated and used in the analyses only when all items were complete. A sensitivity analysis of multiple imputation of missing items indicated no change in conclusions, so we retained only the complete survey data for analysis. Linear mixed effects analysis was conducted separately for each PRO measure to analyze within-subject changes in responses over time. This procedure considers the correlation that occurs for repeated measurements and can handle when the number of assessments is unequal between subjects. All models included fixed effects for time, age, gender, race, and Hispanic ethnicity and an indicator of any CIH use as well as a random intercept. The change in each scale over time did not significantly vary between subjects, so a random effect of time was not included in the models. A *P* value <.05 was used to assess significance. Analyses were performed using SAS software, version 9.4.

Interview notes were analyzed using a directed content analysis approach [30], through inductive open coding [31] to identify themes related to our a priori framework of acceptability, appropriateness, and feasibility [24] that arose through the interviews. Two researchers coded each interview after it took place, coding each set of notes independently and then meeting to identify codes and collapse ideas into broader themes. All coding took place using a discussion and consensus approach, where discrepancies in coding were discussed, evidence was presented, and then a consensus on the coding process occurred. This process continued with each interview, and after the fifth interview, when no new ideas emerged, the researchers determined that saturation had been met, and no additional interviews took place.

## Results

### Overview

Table 1 displays the participant characteristics. The mean age was 54.7 (SD 9.4) years, and the majority were female (66/74, 90%). The majority were white (49/74, 66%) and not Hispanic or Latino (66/74, 89%). Despite disruption in link connectivity access in earlier messages, 74/100 (74%) provided at least one survey response, 53/100 (53%) provided at least two responses, 30/100 (30%) provided at least three responses, 13/100 (13%) provided at least four responses, and 3/100 (3%) provided at least five responses. However, only 2 participants responded to all 6 surveys. Nonresponders were significantly younger than responders (mean 49.0, SD 10.0 years vs 56.7, SD 8.4 years; *P*<.001) and less likely to be white (46.2% vs 66.2%; *P*=.02) but did not differ by gender (female 92.3% vs 89.2%; *P*=.65) or Hispanic ethnicity (12.0% vs 10.8%; *P*=.87).

Response rates were lowest in week 1 (18%) and generally increased in the subsequent weeks (24% for week 2, 34% in week 4, and 33% in week 8). The highest response rate was in the final week of participation, where 42% of participants responded to the survey. This is commensurate with the established expectations of response rates based on our previous work [19,32]. The majority of responses (77.7%) were received within one week with a median of 2 days (25th-75th percentile: 1-5 days) of the SM request to complete the survey.

A total of 74 participants responded to at least one of the survey links sent to them via SM, resulting in 175 responses over the data collection period. Of these, 150 (85.7%) were completed for all 16 items from the 3 scales (PHCS-2, PROMIS-10, and PSS-4). Completeness was lowest for the PROMIS-10 (153/175, 87.4%) and highest for the PHCS-2 (169/175, 96.6%), although the PSS-4 was similar with a completeness of 168/175 (96.0%). A total of 21 surveys were missing between 1 and 14 items across the 3 scales, with the majority missing just 1 item (17/21, 81.0%). There were 2 surveys (1.1%) missing 1 item on the PHCS-2, 16 surveys (9.1%) missing 1 item on the PROMIS-10, and 1 survey (0.57%) missing 1 item on the PSS-4. There was 1 survey missing 2 items on the PROMIS-10 and 4 surveys (2.3%) missing all 16 items from the 3 scales. One additional survey was missing all items for the PROMIS-2, and 2 surveys were missing all items for the PSS-4.

Descriptive statistics of the computed scores from each of the scales are presented in Table 2. Least squares means (SE) for each scale adjusted for age, gender, race, and Hispanic ethnicity are displayed in the table with the corresponding N for each scale (PROMIS-10, PSS-4, PHCS-2; the n values for the mental and physical domains of the PROMIS-10 are the same). In the mixed models, the linear fixed effect of time was not significant for any of the scales, indicating that the scores did not change over time. Age, gender, race, Hispanic ethnicity, and any CIH use were not significant predictors in any of the models.

**Table 1:** Participant characteristics.

Characteristics		Responders (n=74)	Nonresponders (n=26)		*P* value^a^
Age (years), mean (SD)		56.7 (8.4)	49.0 (10.0)		<.001
**Sex, n (%)**					
	Female	66 (89.2)	24 (92.3)		.65
**Race, n (%)**					
	White	49 (66.2)	12 (46.2)		.02
	Black	20 (27.0)	6 (23.1)		—^b^
	Native American	1 (1.4)	0 (0.0)		.02
	Asian	1 (1.4)	2 (7.7)		.02
	Native Hawaiian	1 (1.4)	1 (3.8)		.02
	Multiracial	0 (0.0)	2 (7.7)		.02
	Unknown	2 (2.7)	3 (11.5)		.02
**Ethnicity, n (%)**				.87	
	Not Hispanic or Latino	66 (89.2)	22 (88.0)		
	Hispanic or Latino	8 (10.8)	3 (12.0)		
**Number of surveys completed, n (%)**					—
	1	21 (28.4)	N/A^c^		
	2	23 (31.1)	N/A		
	3	17 (23.0)	N/A		
	4	10 (13.5)	N/A		
	5	1 (1.4)	N/A		
	6	2 (2.7)	N/A		

^a^*P* value from *t* tests, chi square tests, or Fisher exact tests.

^b^Data unavailable.

^c^N/A: not applicable.

**Table 2:** Least squares means of computed scores for each scale per week.

Scale	Week						*P* value
	1 (n=18)	2 (n=24)	3 (n=23)	4 (n=31)	8 (n=31)	12 (n=42)	
PHCS-2^a^, mean (SE)	6.8 (0.36)	6.5 (0.32)	6.6 (0.32)	6.0 (0.28)	6.4 (0.28)	6.6 (0.26)	.22
PROMIS-10^b^ mental, mean (SE)	10.3 (0.56)	11.4 (0.51)	11.1 (0.50)	10.2 (0.46)	10.8 (0.47)	10.4 (0.44)	.09
PROMIS-10 physical, mean (SE)	12.0 (0.47)	11.2 (0.45)	11.3 (0.44)	11.2 (0.40)	11.8 (0.41)	11.3 (0.39)	.17
PSS-4^c^, mean (SE)	7.3 (0.54)	7.4 (0.50)	6.7 (0.50)	7.0 (0.46)	7.4 (0.46)	6.8 (0.44)	.44

^a^PHCS-2: 2-item Perceived Health Competence Scale.

^b^PROMIS-10: 10-item Patient Reported Outcome Measurement Information System.

^c^PSS-4: 4-item Perceived Stress Scale 4.

**Figure 1:**
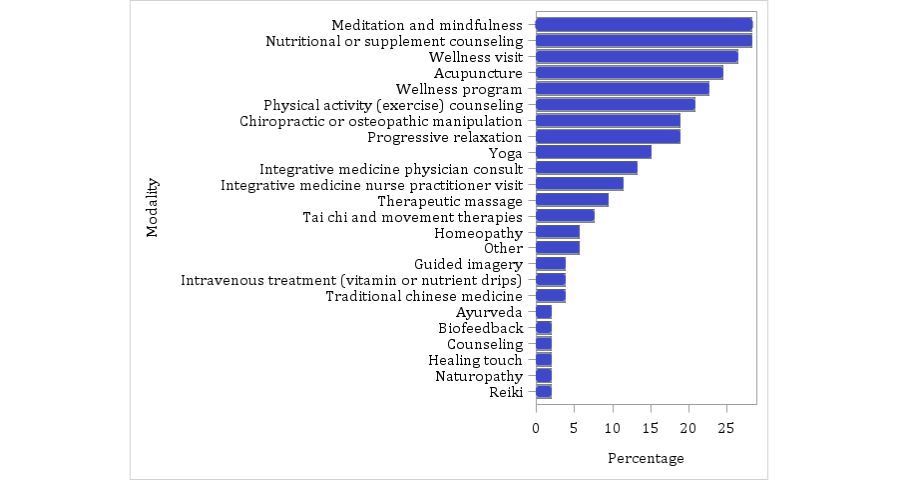
Frequency of reported unique modality use among those who reported use of at least one modality (n=53). The x-axis represents the percentage of patients reporting the modality shown on the y-axis.

Participants also reported their use of CIH modalities during the 12-week period by responding to the question: “Please list the whole health modalities you are currently engaged in. Please check all that apply.” Overall, 53/74 (72%) of respondents reported the use of at least one CIH modality during the 12 weeks. Some participants did not report participating in a CIH program or activity at the time of the project, which may be due to the time lag from participant pool identification and recruitment of individual participants. The reported use of CIH modalities increased over time, with 33/42 (79%) of respondents reporting using at least one modality in week 12, but only 7/18 (39%) of respondents reported using any modality in week 1. One participant reported using 12 different modalities in week 12. Over the 12 weeks, the majority, 33/53 (62%), of those that reported any modality, reported the use of two or more unique modalities (not including reporting of the same modality over subsequent weeks). Figure 1 shows the percentage of unique modality use reported over the 12 weeks (removing duplicate reports for the same modality by individual respondents) among those that reported any modality use. Meditation and mindfulness, nutritional or supplement counseling, wellness visit, acupuncture, wellness program, physical activity (exercise) counseling, progressive relaxation, chiropractic or osteopathic manipulation, yoga, and integrative medicine physician or nurse practitioner visit were the most frequently reported CIH modalities.

### Interviews

Interview data indicated that 4 main themes emerged from our discussions with the PCPs involved in the ePRO implementation: (1) SM can be burdensome for providers (acceptability); (2) PCPs delegate SM duties to their staff, such as registered nurses and licensed practical nurses, to make this process more feasible; (3) staff within the primary care clinic are more appropriate for being involved in the ePRO survey implementation; (4) veteran patients are often challenged with using both SM and the ePRO survey, therefore making this implementation less feasible for them. Table 3 highlights representative quotes from the 5 PCPs mapped to each of these themes.

**Table 3:** Representative quotes from primary care providers.

Themes	Representative quotes
Secure messaging can be burdensome on providers (lack of acceptability)	“For 6 months [I] didn’t have RN so duty fell to me. Crazy and not pleasant. This past year I had a segment of time where RN was not effective so everything came to me anyway. My patients needed a response so I did it.” (physician) “I don’t see it being me. I’m a float and don’t have a panel…We’re busy so I don’t see another 16 secure messages working. It wouldn’t go well for us.” (physician) “I don’t mind once in a while but don’t want to do it from now until I retire…I have no admin time. If there’s any extra admin then I have other things and I won’t be able to do it.” (physician) “If you’re talking about any provider in the clinic doing that right now primary care physicians are completely and totally over the top on what we have to do. Anything you propose as an addition will not be met well.” (physician assistant)
PCPs^a^ delegate secure messaging duties to their staff (RNs^b^ and LPNs^c^; feasibility)	“I’ve had nationally as designated an RN tasked do this [help out with secure messages] so all things are filtered through there.” (physician) “I have my RN and LPN who looks at secure messages for me. LPN takes care of sending out surveys and stuff. She manages the secure messages and she’ll notify me if I need to look at secure messages. She looks for me and sends out to appropriate person. Almost like triaging.” (physician) “Depends on who on the team opens the secure messages. Different teams have different ways. On my team my LPN is very efficient and skilled. She opens messages and knows to go to front clerk or RN. If the message is too long, she comes up to me and says I emailed you and want you to respond to it. She does that well.” (physician) “A pain clinic RN forwards us secure messages, we respond, and she sends it back to patient.” (physician) “My RN gets me the message and many of the messages she answers without talking to me. She’ll write back and ones that RN bumps over to me I see and attend to. And I’ll take care of it from there. On the flip side, I can send secure messages to any of my patients and so can my nurse. Any one of my patients on secure messaging. I can do that as can my nurse.” (physician assistant) “If it’s a test result I feel ok to write to them or if it’s more complicated, I’d write back. If it’s not as complicated, then RN takes care of that. She has some autonomy and many times she writes back, or she asks me could you write to the patient or could you tell me what to say.” (physician assistant)
Staff (RN, LPN, and MSA^d^) would need to send out a survey on patient-reported outcomes (appropriateness)	“Support staff. A well trained MSA could do that easily. At the end of the scheduled visit can send it out.” (physician) “I don’t see it being me. I’m a float and don’t have a panel. A pain clinic RN forwards us secure messages, we respond, and she sends it back to patient.” (physician) “LPN takes care of sending out surveys.” (physician) “If it’s a survey question the RN or LPN or the team clerk could do that.” (physician assistant)
Veterans can face technological challenges (feasibility)	“My veterans who use secure messaging are avid users. Those who don’t use it, who forget and need password; they struggle and that’s the only barrier I see. Non-users won’t be your friends.” (physician) “Some Veterans did not know how to do this [secure messaging] and have difficulties because some Veterans do not have computers at home. Veterans don’t know how to do this.” (physician) “I have quite a few elderly patients who can’t use computers. Smart TV and YouTube are ok but computer is not. Certain populations also have poverty and they don’t have access to a smart phone and don’t want to go to the library for a computer.” (physician)

^a^PCPs: primary care providers.

^b^RNs: registered nurses.

^c^LPNs: licensed practical nurses.

^d^MSA: medical support assistant

## Discussion

### Principal Findings

Using electronic tools for survey administration and dissemination, such as web-based survey platforms, secure email, and text messaging, has the potential to enhance the collection of ePROs by increasing the efficiency of reaching larger populations and collecting data at multiple time points without having to redirect valuable clinical time for data collection. We sought to determine the feasibility of using an SM platform tied to a web-based survey administration via Qualtrics for the collection of ePROs at 6 time points over 12 weeks within the context of VA regulations and systems. Feasibility was assessed qualitatively and quantitatively within the implementation outcomes framework using process evaluation interviews among stakeholders, the duration of time to completion, completeness of responses, and response rates. Overall, of the 100 veterans recruited to participate, 74% (74/100) of participants responded to at least one survey, with 53% (53/100) responding to more than one. However, only 1 participant responded to 5 of the 6 surveys, and only 2 responded to all 6. We learned that, whereas longitudinal ePRO data collection is possible, it is likely that participants will not respond to all surveys requested.

Weekly response rates (Table 2) increased over time, with the highest response rate obtained in week 12 at 42.0% (42/100). This is in keeping with previously published ePRO response rates [20,21,33]. There are a couple of possible explanations for this observation of later surveys achieving higher response rates. First, there was a technical error in survey links sent during the first few weeks of data collection. These technical errors included dead URLs, which decreased response rates. During weeks 6 through 8, a technical error was reported by some participants who called and said they could not open the link. Upon investigation, some users experienced dead short URL links. There were no noted patterns or similarities to participants that called to report such errors. We changed to a universal short URL–generating website. No further calls from participants reporting dead short URLs were received after changing to the short URL–generating site. Such errors clearly impacted the response rates. We learned that ensuring robust testing before the implementation of an ePRO data collection process is extremely important. Survey links were sent weekly for the first 4 weeks. Participant confusion about the completion of multiple surveys and which surveys still needed to be completed may have negatively impacted response rates, particularly when there was short spacing between survey requests. Although the majority of completed surveys were received within 1 week of the SM request, late surveys may have overlapped with subsequent SM requests. The increased response rate in week 12 may, in part, be due to the greater elapsed time between requests.

We found that most surveys (150/175, 85.7%) were complete for all items from the 3 scales, and of those missing items, the majority (17/21, 81%) were only missing 1 item. Although the degree of missing data was small, we learned that this could inform the sample size needed for future research studies. We did not observe a significant change in the computed scores from the scales over the 12 weeks (Table 2). This may be due to a wide variation in modality use among respondents, including that some may have been inconsistent or not using CIH services during the data collection. We did not have baseline measures obtained before the receipt of initial CIH services to evaluate the pre-post effect of CIH on PRO. With a convenience sample size of 100, it is not possible to analyze the effects of individual CIH interventions. Some participants engaged in multiple CIH interventions, making it difficult to isolate the effect of individual interventions. In addition, we learned that the scales used (PHCS-2, PROMIS-10, and PSS-4) may not have been best suited to capture changes in biosocial constructs and whole-person wellness. For example, in the Self-Assessment of Change questionnaire, the 18 paired terms self-assessment measure, assesses not only physical, cognitive, and emotional characteristics but also social, spiritual, and whole-person characteristics. However, the original publication on the PSS-4 [28] indicated that this was a valid measure of stress, and despite being frequently used, recent publications have suggested that the scale may lack internal consistency [34,35]. We learned that future implementation of ePRO data collection should consider utilizing other stress scales.

The small number of participants may also have contributed to the inability to detect any change over time. Only 53 participants reported data at more than one time point. Allowing survey links to *expire* would help to reduce overlap and prevent late responses. Secure messages are available in the MyHealtheVet portal, requiring participants to access the portal to see their messages. Participants who did not access the portal regularly did not receive their messages in a timely manner. In addition, the staff time required to send SM was burdensome. We learned the burden, due to the constant follow-up by the staff to ensure that messages were being received, was a major limitation. The staff had to keep track of when the last survey link was sent using a customized Microsoft Access database and manually send the next link by copying/pasting the Qualtrics auto-generated link into the SM.

It is important to recognize the unique distribution of sex in this participant group. Our group overwhelmingly comprised female veterans. The national veteran population is 10% female [36]. This bias affects the ability to generalize project findings to the broader veteran population. Women tend to be more likely to respond to web-based surveys [37,38]. Our response rate may well have been influenced by our mostly female group. Additionally, female veterans are more likely to participate in CIH activities [39].

This quality improvement project has limitations. First, using a convenience sample may not provide the representativeness that a random sample may provide. Our time from participant pool identification to recruitment was extended, potentially missing opportunities to engage participants. A timelier process for reaching out to the participant pool may have provided a more robust engagement. The personal connection between study teams and participants tends to improve responsiveness [33,38]. Participants did not have in-person contact with the study team. This may decrease responsiveness to surveys [40]. The project experienced several technical glitches (described earlier) that could have been avoided by testing processes before implementation. The project also did not collect data that may have further informed analysis (eg, frequency of computer use to assess comfort with technology use). Future projects may benefit from timely, personal outreach to potential participants, assessment of comfort with technology, and vigorous testing of processes before implementation.

Project findings and lessons learned can inform future research. Future studies should explore the means of dissemination of survey requests that have the capacity for automation to reduce the potential for human and technical errors and reduce workflow burden, which are more efficient for both the staff and participants. In addition, the system should be able to contact participants using their preferred means of communication, whether that is an email address or a text message. Future projects should also examine the optimal spacing of ePRO longitudinal data collection to optimize sensitivity to measures.

### Conclusions

We demonstrated the feasibility of using SM for ePRO data collection among veterans who have received CIH services; although it is possible to use SM for ePROs, in the system’s current state, it is not practical. Lack of automation, workflow burden, and potential for human error make SM a cumbersome system to use when attempting to collect repeated measures from large participant cohorts. Systems that offer customizable features to automate administration on schedule are needed to reduce the provider workflow burden and potential for human error. These results can help inform future studies, including sample size considerations, best practices for workflow and automation, and ideal characteristics for messaging systems.

## Abbreviations

CIH: complementary and integrative health
ePRO: electronic patient-reported outcome
HIT: health information technology
PHCS-2: 2-item Perceived Health Competence Scale
PRO: patient reported outcomes
PROMIS-10: 10-item Patient Reported Outcome Measurement Information System
PSS-4: 4-item Perceived Stress Scale 4
SM: secure messaging
VA: Veteran Administration
VHA: Veterans Health Administration
